# FlyBase 2.0: the next generation

**DOI:** 10.1093/nar/gky1003

**Published:** 2018-10-26

**Authors:** Jim Thurmond, Joshua L Goodman, Victor B Strelets, Helen Attrill, L Sian Gramates, Steven J Marygold, Beverley B Matthews, Gillian Millburn, Giulia Antonazzo, Vitor Trovisco, Thomas C Kaufman, Brian R Calvi, the FlyBase Consortium, Norbert Perrimon, Susan Russo Gelbart, Julie Agapite, Kris Broll, Lynn Crosby, Gilberto dos Santos, David Emmert, L. Sian Gramates, Kathleen Falls, Victoria Jenkins, Beverley Matthews, Carol Sutherland, Christopher Tabone, Pinglei Zhou, Mark Zytkovicz, Nick Brown, Giulia Antonazzo, Helen Attrill, Phani Garapati, Alex Holmes, Aoife Larkin, Steven Marygold, Gillian Millburn, Clare Pilgrim, Vitor Trovisco, Pepe Urbano, Thomas Kaufman, Brian Calvi, Bryon Czoch, Josh Goodman, Victor Strelets, Jim Thurmond, Richard Cripps, Phillip Baker

**Affiliations:** 1 Department of Biology, Indiana University, Bloomington, IN 47408, USA; 2 Department of Physiology, Development and Neuroscience, University of Cambridge, Downing Street, Cambridge CB2 3DY, UK; 3 The Biological Laboratories, Harvard University, 16 Divinity Avenue, Cambridge, MA 02138, USA

## Abstract

FlyBase (flybase.org) is a knowledge base that supports the community of researchers that use the fruit fly, *Drosophila melanogaster*, as a model organism. The FlyBase team curates and organizes a diverse array of genetic, molecular, genomic, and developmental information about *Drosophila*. At the beginning of 2018, ‘FlyBase 2.0’ was released with a significantly improved user interface and new tools. Among these important changes are a new organization of search results into interactive lists or tables (hitlists), enhanced reference lists, and new protein domain graphics. An important new data class called ‘experimental tools’ consolidates information on useful fly strains and other resources related to a specific gene, which significantly enhances the ability of the Drosophila researcher to design and carry out experiments. With the release of FlyBase 2.0, there has also been a restructuring of backend architecture and a continued development of application programming interfaces (APIs) for programmatic access to FlyBase data. In this review, we describe these major new features and functionalities of the FlyBase 2.0 site and how they support the use of *Drosophila* as a model organism for biological discovery and translational research.

## INTRODUCTION

FlyBase (flybase.org) is the principal repository and web portal for genetic data related to *Drosophila melanogaster*, the fruit fly. The FlyBase Consortium is a team of curators, developers, and educators at four sites: Harvard University, University of Cambridge, Indiana University, and University of New Mexico. FlyBase contains data curated from primary scientific literature covering more than a century of genetics research. Over the years, the consortium has developed new formats of data display and new bioinformatic tools to mine these data for biological discovery and translational research. These efforts have transformed FlyBase from a simple database into a powerful *knowledge base*.

The FlyBase site has undergone major changes since our last review two years ago (1). In February 2017, we released a beta version of the next-generation website, which we have named ‘FlyBase 2.0.’ Following a period of public feedback and polishing, FlyBase 2.0 replaced the previous website in December 2017. In this review, we will discuss what is different and better about this next-generation website, and what you can expect from a visit to the new and improved FlyBase 2.0, now and in the future. Although we focus on the new data and tools in this review, there have been some important changes to the FlyBase 2.0 user interface (UI). We refer the interested reader to the previous NAR review in 2017 for an extensive discussion of other aspects of FlyBase (1).

## QuickSearch AND HITLISTS

**Figure 1.**
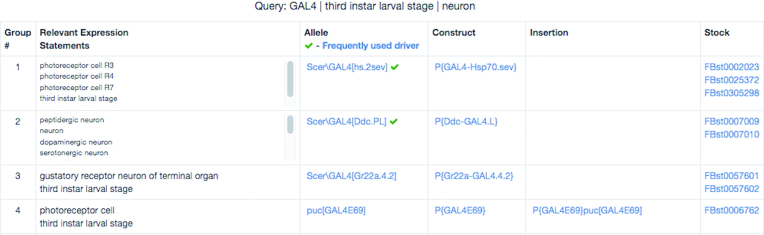
GAL4 Search Result. A result table for a search using the ‘GAL4 etc’ QuickSearch tab, with the ‘integrated table’ output option selected. Cross-references are used to group associated alleles, constructs, insertions and stocks together. Two ‘frequently-used’ GAL4 drivers are flagged.

Usage statistics indicate that most users query FlyBase through ‘QuickSearch’ on the home page. In August of 2017, FlyBase added the ‘GAL4 etc’ tab to ‘QuickSearch.’ This search addressed a long-standing need for a manageable way to search FlyBase for GAL4 and other binary drivers, as well as lacZ and GFP reporters, using different types of expression patterns. The search returns alleles, constructs, insertions, and available stocks, and has an option to display the results in associated groups (Figure 1). It also flags some of the most popular GAL4 drivers based on stock ordering information from the BDSC, and the number of times they are referenced in publications (2). The ‘GAL4 etc’ tab also includes a link to a comprehensive list of these ‘frequently-used’ GAL4 drivers.

**Figure 2.**
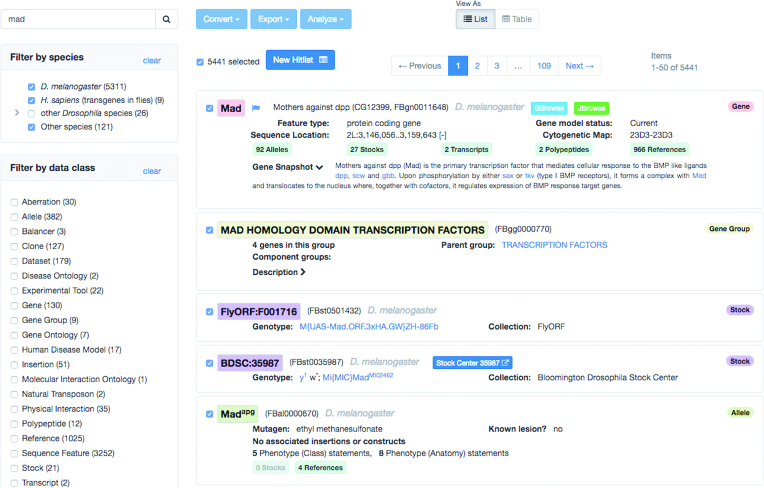
Search Result Hitlist. The result page for Search FlyBase using ‘*Mad*’ as the search term. A ‘hitlist’ is displayed containing genes, stocks, alleles, and many other classes of FlyBase data items (some not shown). The *Mad* gene report button is marked with a blue flag, indicating new annotations in the current release; mousing over the flag shows a summary. The list is framed with an array of tools for filtering by data class and species, pagination, view and analysis.

Although QuickSearch has multiple tabs for specific searches, most people use the generic ‘Search FlyBase’ tab. Given the importance of this entry point, we have devoted much of our effort to fundamentally change and improve the ‘hitlists’ returned by this search for FlyBase 2.0, taking full advantage of the new site architecture (Figure 2). UI improvements to the hitlist result page include a ‘responsive’ layout for viewing on small screens (e.g. smartphones), pagination to reduce loading times, and an embedded new search form.

A significant feature of the new hitlist is that it is ‘mixed’, that is containing all classes of FlyBase data matching the search term. Each matching item is in a panel, containing a concise selection of important information (Figure 2). Color-coded badges along the right margin allow quick scanning of items by data class (Figure 2). A blue flag indicates that new data have been attached to an item in the most recent FlyBase release (Figure 2). Buttons link to FlyBase reports, genome browsers, or new hitlists of related items, e.g. a panel for a given gene will contain buttons for associated alleles, stocks, transcripts, polypeptides and references (Figure 2). Each data class panel also contains class-specific information; for instance an allele panel will display the mutagen used to generate the allele, any associated insertions, and the number of phenotype statements attached to the allele.

The mixed hitlist can be filtered by species or by data class (Figure 2). The species filter lets you choose whether to include/exclude human transgenes in flies, as well as non-*melanogaster* or non-*Drosophila* results. The data class filters can be set to display a more narrow hitlist consisting of a few data classes of interest, or a single data class. Narrowing the search results to a single data class unlocks single-class tools and display options. Note that most of the tabs in the QuickSearch tool generate single-data-class hitlists directly.

**Figure 3.**
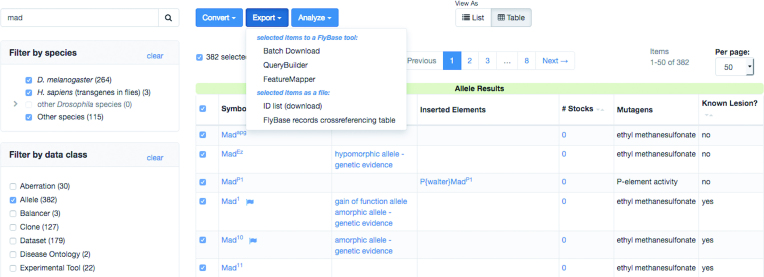
Table View of Search Result Hitlist. The ‘*Mad*’ search result page, filtered to the Allele data class and toggled to table view. The Export tool menu has been expanded.

When the hitlist is filtered to a single data class, a ‘Table’ view option becomes available. The Table view is a vertically compact tabular display, with sortable columns appropriate to that class (Figure 3). A set of analysis tools becomes available when a hitlist comprises a single data class. These tools appear at the top of the hitlist page as a row of buttons labeled ‘Convert,’ ‘Export,’ and ‘Analyze’ (Figure 3). The Convert button is powered by the extensive cross-references between data classes, allowing you to, for instance, turn a list of genes into a list of related references, or a list of alleles into a list of associated insertions. The Export button takes the current hitlist to any of several FlyBase tools, such as Batch Download or Feature Mapper. This is also the best way to download a hitlist as a set of FlyBase IDs. The Analyze button can generate several types of short reports summarizing the hitlist, such as frequencies of anatomy terms or phenotypic classes for an allele hitlist, or can route the hitlist to the Interactions Browser tool. With these enhancements, the hitlist has become a powerful tool for reviewing, refining, and analyzing FlyBase search results.

## REPORT IMPROVEMENTS

**Figure 4.**
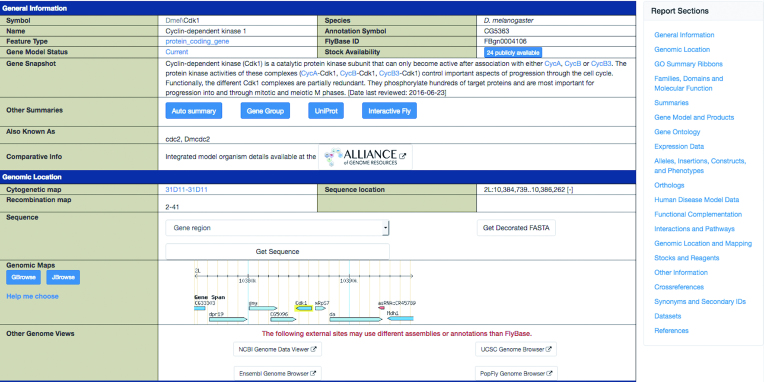
FlyBase Gene Report. FlyBase Gene Report for the *Cdk1* gene. The General Information section serves as a ‘super-summary’ of gene information. The ‘Report Sections’ menu at right floats as the user scrolls in the report, providing an easy navigation tool. The Genomic Location section includes external links to genome browsers at NCBI, Ensembl, UCSC and PopFly.

There have been several notable changes to the FlyBase reports that improve usability and enhance data display. For example, all reports now include a navigation panel on the right hand side of the page (Figure 4). This panel contains links to all the top level sections in the report and can be used to quickly jump to sections of interest. The ‘References’ section of all reports has been improved to make it easier to filter and sort through lists of publications (see the ‘Interactive references and graphical abstracts’ section below for more information).

Summary functional information for genes is important for our site users, especially those involved in translational research. During the last several years, the ‘General Information’ top section of FlyBase Gene Reports has evolved into a ‘super-summary,’ comprising a wide variety of gene overview data (Figure 4). In FlyBase 2.0, this includes a Gene Snapshot, an automatically generated summary, the description of the Gene Group to which the gene belongs (3), UniProt function data, historical Red Book information (4), and a summary from Interactive Fly (http://www.sdbonline.org/fly/aimain/1aahome.htm), whenever these are available. Gene Snapshots are hand-written summaries that are solicited from researchers with expertise in that gene, and provide a quick overview of what is known about that gene's function (1).

**Figure 5.**
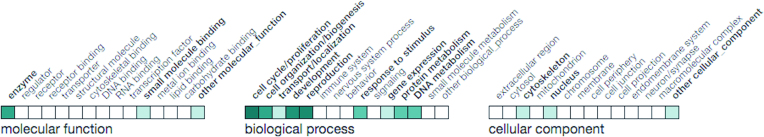
GO Summary Ribbon. GO summary ribbon for *D. melanogaster* gene *Cdk1*, as embedded in a FlyBase Gene Report.

Another useful summary in FlyBase 2.0 Gene Reports is the ‘GO summary ribbon’ (Figure 5). These ribbons were previously implemented at Mouse Genome Database (MGD) (5), and graphically display a top-level distillation of Gene Ontology (GO) terms (6). This ribbon uses the hierarchical structure of the Ontology to condense GO curation down to a few dozen high-level terms, which are then displayed with color intensity chips indicating the number of annotations. More specific terms are displayed as a popup by mousing-over an individual cell, or can be viewed in tabular form in the Gene Ontology section of the report. The GO ribbon significantly enhances the ability of the researcher to quickly assess what is known about a gene’s function.

FlyBase 2.0 Gene Reports now include protein domain graphics from two InterPro data sources, Pfam and SMART, where available (7,8). The Polypeptide Reports display domain information for the specific isoform while the Gene reports display the longest isoform. Mouseover popups and tables show more detailed domain data, and provide links to InterPro reports. These displays complement the tracks in the genome browsers showing this same data aligned to gene models (see below).

## EXPERIMENTAL TOOLS

One indispensable function of FlyBase is as a source of information about fly strains and reagents to design experiments. The importance of this function was highlighted by a 2012 FlyBase survey where ∼90% of respondents said they either find FlyBase ‘very helpful’ or they ‘could not do it [design experiments] without FlyBase.’ To this end, we have created a new ‘Experimental Tool’ data class. Reports describe tools used for gene product detection (e.g. the FLAG tag, EGFP), subcellular targeting (e.g. nuclear localisation signal, signal sequence), expression in a binary system (e.g. UAS, GAL4), or clonal/conditional expression (e.g. FLP, FRT). Each Experimental Tool report provides a description of the tool and its uses, together with browsable tables of related transgenic constructs. These tables list the construct components (e.g. regulatory region, encoded product), transgenic alleles, and constructs, all linked to stocks so that researchers can easily identify useful fly strains. To more easily find these tools, they are also displayed on the relevant allele and construct reports, and the new experimental tool data class has been added to the interactive hitlists. This new experimental tool data class further enhances FlyBase as an important resource for Drosophila research.

## MULTI-SPECIES MINING AND TRANSLATIONAL RESEARCH

For a number of years, FlyBase has hosted data and developed tools to identify orthologs of fly genes in multiple organisms. This has included orthology data from OrthoDB (https://www.orthodb.org/, PMID:27899580) (9) and meta-analysis from DIOPT (https://www.flyrnai.org/cgi-bin/DRSC_orthologs.pl) (10). The OrthoDB orthology calls in FlyBase were updated in 2017, and now include many *Drosophila* species, other insects, and many other species. In addition to links to the orthologous gene, Gene Reports now include links to OrthoDB groups, which allows the user to identify orthologs in up to 5000 species.

DIOPT is a meta-analysis of many different orthology prediction algorithms (including OrthoDB), recently updated in 2018 to include *Arabidopsis thaliana* and three new prediction algorithms. In FlyBase Gene Reports, DIOPT and OrthoDB orthology calls between *Drosophila melanogaster* and a core set of other model organism species are aggregated into a compact display to produce an informative summary. This section also displays links to the protein alignment with the predicted ortholog, and indicates whether the human ortholog, when transferred into *Drosophila*, functionally complements the fly mutant.

FlyBase 2.0 has collaborated with the groups of Norbert Perrimon and Hugo Bellen to develop new online tools that permit searching for orthologous gene function (Gene2Function;http://gene2function.org) (11), conservation of phosphorylation sites and other protein post-translational modifications (https://www.flyrnai.org/tools/iproteindb/web/) (bioRxiv https://doi.org/10.1101/310854), gene interactions across organisms (MIST;http://fgrtools.hms.harvard.edu/mist) (12), and a search tool that returns diverse information about orthologs, human genetics, and disease (MARRVEL;http://marrvel.org) (13). These and other useful links to external resources are featured as icons in the sidebar of the FlyBase home page. These are just a few of the examples of how FlyBase is continuing to collaborate with third parties to develop new tools and support the Drosophila community's foundational discoveries and translational research.

In the last few years, the FlyBase Consortium has increased its participation in The Alliance of Genome Resources (The Alliance;https://alliancegenome.org) (14). The ‘Alliance’ is a collaboration to consolidate and homogenize data presentation across different model organisms, and integrate it with that from humans, to accelerate biological discovery and translational research. The Alliance currently represents the collaboration of six model organism databases (Saccharomyces Genome Database, WormBase, FlyBase, Zebrafish Information Network, Mouse Genome Database, Rat Genome Database) and the Gene Ontology (GO) project. The activities of the Alliance are part of NIH Common Fund's Big Data to Knowledge (https://commonfund.nih.gov/bd2k) Program, an important goal of which is the development of a ‘Data Commons’ (https://commonfund.nih.gov/commons). This Data Commons will be the repository for big data generated by NIH-funded research, with appropriate APIs that ensure that it is accessible to all in a format that is findable, accessible, interoperable, and reusable (FAIR). Over the last two years, FlyBase has provided large data sets to the Data Commons and has developed APIs to facilitate their use. The Data Commons Pilot Phase is part of the NIH Strategic Plan for Data Sciencehttps://www.nih.gov/news-events/news-releases/nih-releases-strategic-plan-data-science to develop new methods for storing, sharing, and analyzing NIH derived datasets in the cloud environment. For more information about these programs, the Alliance, and FlyBase's role in them, we refer the reader to a recent comprehensive review (14).

## INTERACTIVE REFERENCES AND GRAPHICAL ABSTRACTS

Nearly all FlyBase report pages have a ‘References’ section that contains a list of publications associated with the given entity (gene, allele, insertion, etc.). This section has been enhanced in FlyBase 2.0 with an interactive sidebar that allows the user to filter by publication type, e.g. ‘research paper’ or ‘review’ (Figure 6). Users can also sort by year or author, search by text, and export edited publication lists to Batch Download, as a HitList, or as RIS citations for their favorite reference manager. For the Gene Report, one of the increasing challenges is distinguishing between papers that focus on a gene from those that have only a minor reference to it, for example as one data point in a genome-wide analysis. To help the user identify the papers most relevant to that gene, we have introduced a ‘representative publication’ section. This category contains up to 25 papers that FlyBase has identified as most informative with regard to the identification and function of a particular gene. To identify these representative publications, we developed an algorithm that ranks papers by relevance, based on the amount and nature of data curated for the given gene, especially prioritizing papers that mention the gene in the title or abstract. The ability to identify the most informative papers among the hundreds that mention a gene, together with the other sorting capabilities of the reference section, begins to address the problem of grappling with the rapidly-growing biological literature.

**Figure 6.**
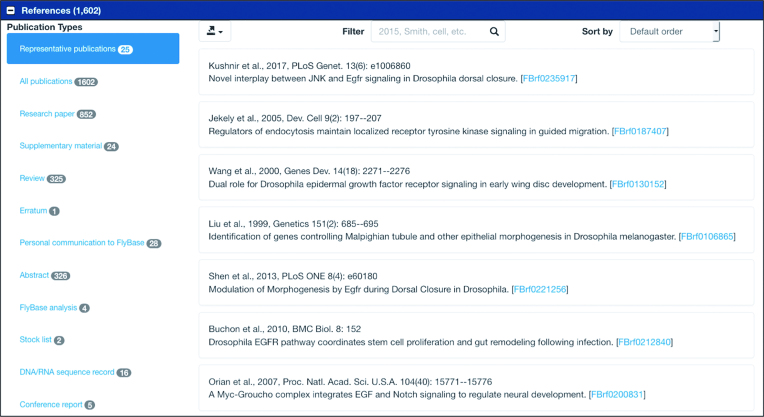
Interactive References Section. References section with options for filtering by publication types (left sidebar) including representative publications, and various sort, search, and export options.

Another way that FlyBase is trying to help users find the relevant literature is the inclusion of ‘graphical abstracts’ - images that summarize the findings of a paper, first introduced by Cell Press a number of years ago. FlyBase has entered into an agreement with Cell Press to display the graphical abstracts in the corresponding reference report. Thumbnails of these graphical abstracts are also included in panels for reference hitlist items, when available. Clicking on the graphical abstract directs the user to the abstract and paper at Cell Press.

## NEW GENOME BROWSER TRACKS AND MIGRATION FROM GBrowse To JBrowse

For a number of years, the GBrowse genome browser in FlyBase has displayed annotated gene models and many other mapped features of the genome and epigenome, all shown as separate ‘tracks’ (15) Tracks unique to FlyBase include signal graphs of RNA-Seq from different projects over developmental time or in response to environmental stimuli and protein domains aligned to the *D. melanogaster* genome reference strain (1). Protein domain information has been enhanced with a new track that shows domains predicted by SMART, supplementing the previously implemented ‘Pfam’ track, and providing a second independent view of which protein domains are encoded by a gene and how they are distributed among exons (7,8). Gene and Polypeptide Reports also contain schematics of these domains (see **Report improvements**, above).

While GBrowse has been the FlyBase genome browser platform for many years, with FlyBase 2.0 we have begun migrating genome tracks to a next-generation genome browser called JBrowse (16). JBrowse has a number of unique features that improve genome browsing ease and functionality, such as greater speed and responsiveness, configurable tracks, same-screen track selection, and click-and-drag navigation. Most pages with genome browser links in FlyBase 2.0 currently allow users to select between GBrowse and JBrowse. Once our migration to JBrowse is complete, GBrowse will be deprecated but still accessible for a year, after which JBrowse will be the only genome browser hosted by FlyBase. In addition to the genome browsers on FlyBase, we have recently added links within the ‘other genome views’ section of the Gene Report to browsers at NCBI, Ensembl, UCSC, and PopFly, which have different annotations and functionalities (Figure 4). For example, the PopFly browser depicts DNA polymorphisms identified in natural populations of *D. melanogaster*. FlyBase continually evaluates new community data sets for inclusion into our genome browsers. Current plans include improvements to the developmental proteome annotation and adding locations of efficient gRNA target sites for CRISPR engineering that have been predicted by the Drsosophila RNAi Screening Center (DRSC) (https://fgr.hms.harvard.edu/) (17).

## NEW TOOLS FOR POWER USERS

The building of FlyBase 2.0 entailed a significant change to backend architecture that enabled new capabilities for ‘power users’. We improved cloud compatibility, added an application programming interface (API) (https://flybase.github.io/), and fundamentally reorganized the code to have a more modular structure. We continue to support a publicly accessible Chado database (https://flybase.github.io/) and downloads of XML, FASTA, GFF, GTF, and other bulk data files via our FTP site (ftp://ftp.flybase.org/).

## CONNECTIONS TO THE COMMUNITY

FlyBase greatly benefits from a well-engaged user community. Since 2014, the FlyBase Community Advisory Group (FCAG), a group of over 500 researchers world-wide with a commitment to improving FlyBase, have responded to regular surveys with invaluable information about how researchers actually use FlyBase, and suggestions for new capabilities. This feedback continues to shape how FlyBase adapts to new data and user needs. Our goal is to have a representative in FCAG from every Drosophila lab; new representatives can register by following the FlyBase Community Advisory Group link under the Community menu on FlyBase (http://flybase.org/wiki/FlyBase:Community_Advisory_Group). Another continuing effort is the production of video tutorials, which has accelerated in the last two years with eight new videos posted to our YouTube channel (https://www.youtube.com/c/FlyBaseTV), covering various searching techniques, new features of the FlyBase 2.0 website, and JBrowse. The new website also displays the FlyBase Twitter feed (https://twitter.com/FlyBaseDotOrg) on the left sidebar of the homepage, which we use to alert users of new data and features and of topical news relevant to the fly community.

## LOOKING TO THE FUTURE

A future challenge will be to keep up with the accelerating growth of biological information, including the ever-increasing amount of big data from new high-throughput methods. Among these new methods are single-cell RNA sequencing (RNA-Seq), which yields volumes of fine-grained temporal and spatial information about gene expression. To realize the full potential of this method, it will be imperative to develop new approaches to integrate and display the large amount of data in an interactive format that is both useful and facile. FlyBase will continue to integrate developmental proteome data as it becomes available, and integrate it with RNA-Seq data via graphical displays and JBrowse to produce a powerful tool for functional genomics. Future development of new interactive displays for pathways and interactions among these gene products will further empower a systems approach to understanding cellular networks. We also envision the integration of other fundamentally new data classes. Among these are Drosophila metabolic pathways and the microbiome, the population of microorganisms in and on the fly. Given that the construction of FlyBase and other MODs has been gene-centric, integrating these data will present new challenges, and will require third party collaborations and link-outs. Of course, meeting all of these challenges of growing biological information will depend on the availability of sufficient resources.

FlyBase will also continue as an active member of the the Alliance of Genome Resources (The Alliance; https://alliancegenome.org) (14). This will include efforts to homogenize data and develop new displays and tools for foundational and translational research. Part of these efforts will be creation of new APIs that allow power users to retrieve and work with big data sets deposited in the NIH Data Commons. These will be important future efforts as the torrent of big data and the importance of bioinformatics for biomedical research continues to increase.

Over the last 27 years FlyBase has evolved from a simple database into a powerful knowledge base. In addition to its essential role of curating and disseminating fly data, FlyBase is continuing to develop new tools for discovery of gene function across organisms and their links to human disease (18). FlyBase remains essential to support the numerous data types specific to the fly research community so that the full potential of *Drosophila* for biological discovery and translational research can be realized (19). Continuing to build on the FlyBase 2.0 knowledge base will further empower the Drosophila community to explore new ideas, to seek out new aspects of life, and to boldly go where no one has gone before.
